# Correlation between structural determinants and universal health coverage in 2010 and 2019: An analysis of the global burden of disease study

**DOI:** 10.1371/journal.pgph.0004770

**Published:** 2025-07-11

**Authors:** Orlando Luiz do Amaral Junior, Maria Laura Braccini Fagundes, Fernando Neves Hugo, Nicholas J. Kassebaum, Jessye Melgarejo do Amaral Giordani

**Affiliations:** 1 Postgraduate Program in Dental Sciences, Federal University of Santa Maria, Santa Maria, Brazil; 2 College of Dentistry, Department of Epidemiology and Health Promotion, New York University, New York, New York, United States of America; 3 Institute for Health Metrics and Evaluation, University of Washington, Seattle, Washington, United States of America; University of Essex, UNITED KINGDOM OF GREAT BRITAIN AND NORTHERN IRELAND

## Abstract

**Objective:**

To assess the correlation between structural determinants - governance, macroeconomic policies, culture/societal values, and public and social policies - and the Universal Health Coverage (UHC) Effective Coverage Index in 2010 and 2019, in 204 countries and territories.

**Methods:**

This ecological study analyzed UHC effective coverage in 204 countries and territories using estimates from the Global Burden of Disease (GBD) 2019 study. Structural determinants were examined across five dimensions. Spearman’s correlation was used to examine the correlations.

**Results:**

UHC showed a positive correlation with structural determinants in both years. In 2010, moderate correlations were observed for governance (ρ = 0.61), GDP (ρ = 0.69), SDI (ρ = 0.62), and government expenditure (ρ = 0.58). In 2019, governance (ρ = 0.56), GDP (ρ = 0.71), SDI (ρ = **0.66),** and government expenditure (ρ = **0.48)** remained significantly correlated with UHC. GDP and SDI consistently showed the strongest correlations in both periods.

**Conclusion:**

Countries with more favorable structural conditions had greater UHC, emphasizing the influence of governance and socioeconomic context on health systems performance. Persistent disparities highlight the need for policies targeting social and economic inequalities to achieve universal health coverage globally.

## Introduction

Health is a fundamental human right and a central objective of any health system [1]. An effective health system guarantees equitable access to high-quality services across the continuum of care, including health promotion, disease prevention, treatment, and rehabilitation [1]. Universal Health Coverage (UHC), a key target of the post-2015 sustainable development agenda, aims to ensure the fair allocation of resources to improve population health [2]. UHC guarantees access to necessary healthcare without imposing financial hardship, serving as a critical strategy to advance health equity and reduce disparities [3]. Achieving UHC is essential for promoting health equity and overall well-being and ensuring that no one is left behind [4].

Focusing on Universal Health Coverage (UHC) is crucial for assessing how close populations are to achieving optimal health outcomes [1]. Despite its relevance, limited research has explored the structural determinants associated with UHC [2,5]. Most studies have concentrated on technical aspects, often overlooking the broader political and economic contexts that shape the adoption, implementation, and sustainability of health reforms [3,4]. The success of UHC is intrinsically linked to factors such as political commitment, economic conditions, and the development of social and public policies. [3,4].

Although evidence indicates that structural determinants of health significantly influence Universal Health Coverage (UHC) outcomes, research in this area remains scarce [5]. Additionally, no global studies to date have examined UHC in relation to the five dimensions of the World Health Organization’s structural framework: governance, macroeconomic policies, culture/social values, and public and social policies [6,7]. This gap highlights the need for research that examines the broader political, social, and economic contexts shaping the implementation of UHC across different countries [4,8] Expanding this knowledge is essential to identify effective strategies for overcoming context-specific challenges and advancing progress toward UHC goals [5,9].

In summary, examining the broader political and economic dynamics that impact UHC is fundamental to advancing health equity and improving outcomes across populations. Therefore, this research could enhance policymakers’ and health professionals’ understanding of the systemic barriers to equitable healthcare access and to support the global advancement of UHC [1,4]. By addressing key structural determinants, governments and organizations can promote high-quality health care for all without financial hardship. To contribute to this field, this study investigates the association between the structural factors, including governance, macroeconomic policies, culture/societal values and public and social policies, and the UHC Effective Coverage Index across 204 countries and territories in 2010 and 2019. By adopting a global perspective, the study seeks to provide a comprehensive understanding of the structural determinants of UHC and to inform strategies that foster equitable access to health services worldwide.

## Methods

### Outcome

The outcome measure in this study is the UHC Effective Coverage Index, a metric developed by the Institute for Health Metrics and Evaluation, which provides a standardized approach to assess how health systems deliver services while accounting for service availability and quality of care [1]. The UHC Effective Coverage Index provides a summary measure, with component metrics and domain-specific scores revealing patterns of health system performance.

The construction of the UHC integrates 23 metrics spanning five domains of healthcare delivery: health promotion, disease prevention, treatment services, rehabilitation and palliative care. The framework captures aspects of health system performance, from prevention to treatment interventions [1]. The index incorporates five population age groups: reproductive, maternal and newborn health; children under five years; children and adolescents aged 5–19 years; adults aged 20–64 years; and older adults aged 65 years and above. This stratification allows assessment of how health systems meet the needs of different demographic groups [1].

The 23 metrics represent two dimensions of health system performance. Direct coverage measures, such as the proportion of births attended by skilled health personnel or antiretroviral therapy coverage rates, provide evidence of service utilization. Outcome-based indicators, like mortality-to-incidence ratios for cancers, serve as proxies for assessing quality of care [1]. The calculation methodology involves technical steps. Each indicator is normalized to a 0–100 scale, where 0 represents the lowest observed performance globally and 100 represents either a target or the highest observed performance. This standardization enables comparisons across metrics and between countries and time periods [1].

Health gain weights are applied to each metric. These weights reflect potential population health benefits achievable through optimal service delivery, estimated through analysis of disease burden data and intervention effectiveness. The weighting process considers the targeted disease burden, measured in disability-adjusted life years (DALYs), and the effectiveness of the intervention in reducing this burden. Interventions are classified into four effectiveness categories based on their expected impact. The aggregation of weighted indicators follows a protocol [1]. The methodology employs a weighted geometric mean approach, ensuring improvements in weaker areas of performance contribute to the overall index score. Aggregation occurs in two stages: within age groups to create age-specific coverage estimates, and across age groups using demographic weights to produce a national-level index. Validation procedures are incorporated to ensure robustness [1]. These include cross-validation with other health system performance metrics, sensitivity analyses to test alternative weighting schemes, and statistical adjustments for variations in data quality. Missing data are handled through imputation techniques that maintain the integrity of cross-country comparisons. The data sources include the core GBD 2019 estimates, household surveys, national health information systems, disease registries, and surveillance systems. This multi-source approach mitigates limitations of single data sources while providing coverage of health system dimensions [1]. The development of this index contributes to global health measurement, providing policymakers with a tool to track progress toward universal health coverage across countries and over time. Further details on UHC Effective Coverage Index development are published elsewhere [1].

### Exposure variables

#### Macroeconomic policy.

The macroeconomic policy indicator used in this study was the gross domestic product per capita, also known as GDP, which provides an estimate of the total value of goods and services produced in a country during a given period. GDP is calculated annually and is commonly used to assess a country’s economic growth metrics [10]. The calculation is based on GDP per capita, which is measured in the country’s respective currency and then converted into international dollars. Additional information on GDP calculation can be found on the World Bank’s website (http://microdata.worldbank.org/index.php/home).

GDP represents a country’s income in terms of equivalent purchasing power, accounting for differences in the cost of living across countries. As an indicator of the flow of new final goods and services produced during a period, a country’s GDP will be zero if it does not produce anything in a year. Data on the GDP of each country included in the study was collected from the World Development Indicators (WDI), a collection of development indicators compiled by the World Bank from officially recognized international sources. The WDI is considered to be one of the most current and accurate sources of global development estimates, providing national, regional, and global estimates [11]. The GDP variable will be categorized into “low”, “medium”, or “high” based on previously established cut-off points [10,12].

#### Public policy.

To estimate public policies, a metric of health expenditure was used. It includes direct expenditures aimed at improving the health status of the population and/or distributing medical goods and services to the population [13]. For this study, a World Bank metric was utilized, specifically health spending as a proportion (%) of government spending. This variable was chosen due to its relevance in reflecting the priority given by governments to health policies and the availability of the data in the World Bank database, which is compiled from officially recognized international sources. The variable was then categorized into tertiles of “high”, “medium”, and “low” spending to facilitate the interpretation of health inequalities.

A higher percentage of health spending as a proportion of government spending implies greater investment in healthcare and greater commitment to improving the health of the population. More detailed information on this metric is available at: https://data.worldbank.org/indicator/SH.XPD.PUBL.GX.ZS?view=chart [12].

#### Governance.

Six dimensions of governance are evaluated for 204 economies using 32 individual data sources from various research institutes, international organizations, non-governmental agencies, and private sector companies, as detailed in Kaufmann et al. (2010) [14]. These dimensions include: Voice and Accountability, Political Stability and Absence of Violence/Terrorism, Government Effectiveness, Regulatory Quality, Rule of Law, and Control of Corruption. The sources used for each dimension are thoroughly explained in the original reference, providing a comprehensive methodology for the assessment [14], as follows:

- Voice and Accountability: This dimension measures citizens’ perceptions of the extent to which they can participate in choosing their government, freedom of expression, freedom of association, and free media.
- Political Stability and Absence of Violence/Terrorism: This dimension measures perceptions of the likelihood of political instability and/or politically motivated violence.
- Government Effectiveness: This dimension measures perceptions of the quality of public services, civil services and the degree of its independence from political pressures, quality of policy formulation and implementation, and credibility of the government’s commitment to policy.
- Quality of the Regulator: This dimension measures the government’s ability to formulate and implement policy, and strong regulations that allow and promote the development of the private sector.
- Rule of Law: This dimension measures the degree to which agents trust and comply with the rules of society, including the quality of the execution of contracts, property rights, police and the courts, as well as the likelihood of crime and violence.
- Control of Corruption: This dimension measures the extent to which public power is exercised for private gain, including minor and major forms of corruption, as well as state “capture” by elites and private interests.

The Governance Indicators Framework produces a percentage across all countries, from 0 (lowest) to 100 (highest). This composite metric is commonly used in research to explore the relationship between governance and various socio-economic outcomes, including health [12,14].

#### Social policy.

The Sociodemographic Index (SDI) is a comprehensive metric developed by the GBD 2015 collaborative group (Institute for Health Metrics and Evaluation 2016) that serves as a determinant for measuring social policy. Unlike a binary economic metrics such as “developed/developing”, the SDI is an attempt to provide a more nuanced understanding of a country’s level of development by incorporating multiple dimensions of sociodemographic factors. Specifically, the SDI is calculated as the geometric mean of three rescaled components: average income, average schooling of individuals aged 15 years or older, and the number of children per woman aged 10–24 years. SDI scores range from 0 (representing the lowest values) to 1 (representing the highest values) [15,16].

To classify countries, the SDI scores are then used to create quintiles based on previous published studies. This approach ensures that countries are categorized in a way that reflects the most current and comprehensive understanding of sociodemographic factors and facilitates cross-country comparisons. Overall, the SDI is a robust and widely used indicator that captures multiple dimensions of development and is essential for measuring and evaluating the impact of social policies on populations [15,16].

### Culture/Societal values

The percentage of women holding seats in national parliaments is determined by calculating the number of seats occupied by women members in the single or lower chambers of these parliaments and expressing it as a percentage of the total number of occupied seats. This calculation involves dividing the total number of seats occupied by women by the overall number of seats in parliament. National parliaments can be bicameral or unicameral. This indicator covers the single chamber in unicameral parliaments and the lower chamber in bicameral parliaments. It does not cover the upper chamber of bicameral parliaments. The number of countries covered varies with suspensions or dissolutions of parliaments. Parliaments vary considerably in their internal workings and procedures, however, generally legislate, oversee government and represent the electorate [17]. In terms of measuring women’s contribution to political decision making, this indicator may not be sufficient because some women may face obstacles in fully and efficiently carrying out their parliamentary mandate. Even so, the percentage of women in national parliaments is considered a relevant measure of societal values, particularly in terms of gender equality and political inclusion, which are central aspects of cultural and societal structures in a given society [18,19]. This variable was categorized into tertiles to facilitate the understanding of the data distribution. (available at: https://data.worldbank.org/indicator/SG.GEN.PARL.ZS).

### Ethical aspects

This study exclusively utilized secondary, anonymized data from the Global Burden of Disease (GBD) study and the World Bank database, both of which are publicly available and do not require ethical approval. According to the policies of the Institute for Health Metrics and Evaluation (IHME), which oversees the GBD study, all data are aggregated and anonymized to ensure the protection of individual privacy and compliance with ethical standards. Similarly, the World Bank database provides open access to global development indicators and anonymized datasets for public use. For more information on the data policies and ethical considerations of these sources, please visit the IHME website: https://www.healthdata.org and the World Bank Open Data website: https://data.worldbank.org.

### Statistical analysis

**Fig 1:**
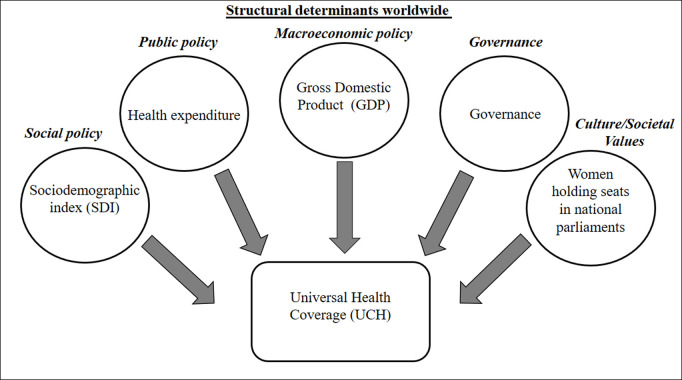
Theoretical conceptual model seeking to understand the correlation between structural determinants and universal health coverage.

The data analysis was conducted using STATA 14.0 software (Stata Corporation, College Station, TX, USA). In the initial stage of this study, a descriptive analysis was conducted to ascertain the estimated results of each variable concerning universal health coverage. The proposed conceptual model sought to follow the theoretical paths that discuss the structural determinants, relying heavily on studies carried out previously [5,9] (Fig 1). Moreover, a decision was made to incorporate a categorical analysis to enhance the visualization of data. Subsequently, the association between UHC and the exposure variables was examined using Spearman correlation, which was chosen due to the non-parametric distribution of data [20]. The results were presented along with their respective 95% confidence intervals (PR; 95% confidence interval [CI]), and a significance level of 5% (p < 0.05) was adopted. All analyses were conducted separately for each year (from 2010 and 2019). Both predictors and outcome variables were stratified independently for each period to ensure comparability within the respective time frames. While the predictors presented in Table 1 were categorized to facilitate interpretation and the identification of potential patterns or inequalities, in the correlation analysis, these variables were used in their continuous form to preserve the integrity of the statistical associations.

**Table 1:** Mean Universal Health Coverage (UCH) by structural determinants worldwide in 2010 and 2019.

Structural determinants	UHC Coverage Average in 2010		N	UHC Coverage Average in 2019		N
	Mean	IC95%^*^		Mean	IC95%^*^	
**Gross Domestic Product (GDP)**						
Low	43.0	39.1 – 46.8	90	49.5	47.5 – 51.51	70
Medium	65.6	59.3 – 71.9	21	52.9	26.9 – 79.0	6
High	74.4	71.8 – 77.0	101	72.2	69.1 – 75.3	104
**Health expenditure (Tertile)**						
T1	50.3	42.4 – 58.2	56	52.2	46.8 – 57.7	81
T2	57.0	54.2 – 59.9	78	49.1	45.5 – 52.7	47
T3	73.8	67.8 – 79.9	48	77.7	73.0 – 82.4	52
**Governance**						
Low	35.5	28.7 – 42.3	45	37.3	31.0 – 43.6	46
Medium	58.0	55.8 – 60.2	89	58.0	55.1 – 60.8	87
High	76.7	73.4 – 80.1	80	75.9	71.0 – 80.7	62
**Sociodemographic index (SDI) (quintiles)**						
Q1	34.2	27.4 – 41.0	43	34.0	27.9 – 41.8	40
Q2	51.8	48.0 – 55.5	43	59.3	54.8 – 63.9	70
Q3	70.6	66.2 – 75.1	47	62.0	53.28 – 70.7	7
Q4	63.2	60.4 – 66.0	33	64.4	61.1 – 67.7	44
Q5	75.7	72.5 – 78.8	60	74.8	70.8 – 78.8	58
**Women holding seats in national parliaments (Tertile)**						
T1	53.5	50.8 – 56.1	51	56.7	53.0 – 60.4	49
T2	63.3	57.5 – 69.1	50	46.4	39.8 – 52.9	64
T3	47.0	42.3 – 51.7	91	69.5	65.5 – 73.5	81

* =95% Confidence Interval.

## Results

Table 1 shows differences in the mean values of Universal Health Coverage (UHC) among countries with different levels of Gross Domestic Product (GDP), healthcare expenditure, governance, and Social Development Index (SDI). The results suggested persistent inequalities in both studied periods. Low GDP countries had a mean UHC of 49.5% in 2019, while high GDP countries had a mean of 72.2%. Inequality is also observed in healthcare expenditure, where the first and third tertiles had means of 52.2% and 77.7%, respectively. In terms of governance, countries with poor governance had a mean UHC of 37.3%, while countries with good governance had a mean of 75.9%. The SDI showed a mean UHC of 34.0% in the poorest quintile (Q1) in 2019 and 74.8% in the richest quintile (Q5).

Regarding the variable of women in national parliaments in 2019, countries with higher female participation had an average of 69.5%, while countries with lower female participation had an average of 56.7%. These results highlight persistent inequalities in access to Universal Health Coverage (UHC) among countries, which have remained in both 2010 and 2019.

In Table 2, the results of Spearman’s correlation between UHC and structural determinants for the years 2010 and 2019 are presented. It was observed that for the period of 2010, the correlation was moderate, with coefficients of 0.61 for governance, 0.69 for GDP, 0.62 for SDI, and 0.58 for government expenditure. For the period of 2019, the determinants of governance and GDP showed a correlation, with coefficients of 0.56 and 0.71 to SDI, respectively, and government expenditure, with coefficients of 0.58 and 0.48, respectively. The absolute magnitude of the observed Spearman correlation coefficient can be interpreted as follows: a range of 0.10-0.39 indicates a weak correlation, 0.40-0.69 indicates a moderate correlation, and 0.70-0.89 indicates a strong correlation. The interpretation of the findings was based on the previous literature [21].

**Table 2:** Spearman correlation between universal health coverage and structural determinants in 2010 and 2019 respectively.

Structural Determinants	2010 (Year)						2019 (Year)					
	UHC	Governance	GDP	SDI	Gov. Expenditure	Women in national parliament	UHC	Governance	GDP	SDI	Gov. Expenditure	Women in national parliament
Universal Health Coverage (UHC)	1.00						1.00					
Governance	**0.61** ^*^	1.00					**0.56** ^*^	1.00				
GDP	**0.69** ^*^	0.87	1.00				**0.71** ^**^	0.77	1.00			
SDI	**0.62** ^*^	0.83	0.84	1.00			**0.64** ^*^	0.70	0.88	1.00		
Government expenditure	**0.58** ^*^	0.59	0.39	0.43	1.00		**0.58** ^*^	0.59	0.58	0.47	1.00	
Women in national parliament	0.18	0.12	0.10	0.1	0.19	1.00	0.26	0.23	0.27	0.21	0.29	1.00

* =Moderate correlation/

** =Strong correlation.

## Discussion

This study aimed to examine the relationship between Universal Health Coverage (UHC) and key structural factors, governance, macroeconomic policies, and public and social policies across countries in 2010 and 2019. The findings indicate that higher per capita GDP, greater health expenditure, elevated Socio-demographic Index (SDI) scores, and stronger governance are positively associated with better UHC Effective Coverage. These results contribute to a broader understanding of the structural drivers of UHC, moving beyond the traditional emphasis on income and health outcomes. The observed associations highlight the relevance of these determinants in shaping health policy and ensuring the delivery of high-quality health services.

Indicators such as GDP, SDI, governance, and government spending are important for monitoring development, evaluating public policies, and informing strategies aimed at achieving universal access to healthcare [3,4]. These metrics provide valuable information into a country’s economic and social conditions, helping to identify priority areas for investment to improve health outcomes. By monitoring these indicators, policymakers can make evidence-based decisions on resource allocation to ensure equitable access to quality healthcare services, regardless of socioeconomic status [3,4]. Incorporatin these structural factors into health policy formulation is vital for improving population health and achieving Universal Health Coverage in alignment with the United Nations Sustainable Development Goals [22,23]. It is imperative that political leaders and policymakers recognize the impact of these factors and foster conditions that promote health equity, population well-being, and the long-term sustainability of health systems [24].

The observed correlation between governance and Universal Health Coverage (UHC) represents a significant and novel contribution to the current framework employed by the Global Burden of Disease (GBD) study [22,25]. These findings reinforce the need for a comprehensive approach to improving health outcomes one that goes beyond healthcare infrastructure to include social determinants of health and the effective implementation of health policies [22]. Empirical evidence strongly supports the role of good governance as a fundamental determinant for achieving UHC. This underscores the importance of not only investing in healthcare services but also establishing robust governance structures within health systems. Policymakers should prioritize institutional transparency, encourage participatory governance in health decision-making, and implement accountability mechanisms to monitor the equitable and efficient use of health resources [4,26]. Additionally, macroeconomic policies must emphasize sustained investment in health, particularly in low- and middle-income countries where chronic underfunding persists. Key strategies include increasing public health expenditure relative to GDP, adopting progressive taxation to finance healthcare, and advancing multisectoral policies that integrate health with social protection and education initiatives, measures essential to advancing UHC and reducing global health inequities [26].

An important finding of this study is that, although correlations between structural factors and the UHC Effective Coverage Index were observed in both 2010 and 2019, there was a notable increase in health expenditures and UHC coverage over the period. improvements in governance, macroeconomic conditions, and public and social policies may have contributed to the global expansion of UHC, potentially explaining the stronger correlations observed in 2019. These results reinforce the notion that sustained investment and structural reforms play a critical role in advancing equitable access to healthcare.

Although the result indicate no strong correlation between the proportion of women in parliament and Universal Health Coverage (UHC), previous studies suggest that women’s political representation can influence a range of health outcomes, including maternal and reproductive health, gender-based violence, mental health, and access to health services [19,27]. One possible explanation for this discrepancy is that the relationship between women’s representation and UHC may be mediated by institutional and political factors not accounted for in this study, such as the actual policymaking power held by female legislators, the degree of gender mainstreaming in national health policies, and the broader political culture. Moreover, while the proportion of women in parliament serves as a proxy for gender representation, it does not directly measure the extent to which gender-sensitive health policies are implemented or the degree to which female policymakers advocate for health equity [28]. Future research should investigate these complexities using more detailed governance indicators and policy-tracking methodologies. Women’s political participation can promote greater attention to gender-related health issues, influence resource allocation, and support the development of inclusive health policies. Furthermore, increasing women’s representation in parliament may help challenge gender norms and stereotypes that contribute to health disparities and discrimination within healthcare systems [27].

Despite notable progress, substantial challenges remain in the pursuit of comprehensive Universal Health Coverage (UHC) [22,29]. Studies indicate a persistent lack of adequate health investment in many countries, particularly in low- and middle-income settings [8]. There is an urgent need to expand investments aimed at strengthening healthcare infrastructure, training health professionals, and ensuring the provision of accessible, high-quality services. In addition, persistent inequalities in health coverage continue to pose a major obstacle [30]. While UHC has advanced in several regions, significant disparities remain across socioeconomic and geographic groups, leaving many individuals—especially those in remote or marginalized communities—without the essential health services they require [5,8].

This study presents some limitations. Although the ecological design is valuable for identifying correlations between structural factors and the UHC Effective Coverage Index, it does not allow causal inferences. The risk of ecological fallacy is inherent, as correlations identified at the population level may not reflect individual-level relationships. Moreover, the cross-sectional nature of the analysis limits the ability to capture temporal dynamics; structural changes in governance, macroeconomic conditions, and public policies often require extended periods to influence health outcomes, which may not be evident in a single time frame. The use of Spearman’s correlation, while appropriate for exploratory purposes, does not establish causality and should be interpreted with caution. Additionally, reliance on the UHC Effective Coverage Index as a proxy for health coverage may not fully reflect other critical dimensions of healthcare quality, such as service effectiveness and patient satisfaction. It is also important to acknowledge that unmeasured variables, such as cultural contexts and specific political and economic conditions, may have influenced the findings. Despite these limitations, the study significantly contributes to understanding the factors associated with universal health coverage.

In conclusion, the findings suggest that structural determinants are correlated with Universal Health Coverage (UHC) and should be systematically considered in the development of health policies. Incorporating these factors into policy planning is essential to improving population health, promoting health equity, and ensuring the long-term sustainability of healthcare systems.
